# Sperm RNA Payload: Implications for Intergenerational Epigenetic Inheritance

**DOI:** 10.3390/ijms24065889

**Published:** 2023-03-20

**Authors:** Simeiyun Liu, Upasna Sharma

**Affiliations:** Department of Molecular, Cell and Developmental Biology, University of California, Santa Cruz, CA 95064, USA

**Keywords:** small RNAs, epigenetics, inheritance, sperm

## Abstract

There is mounting evidence that ancestral life experiences and environment can influence phenotypes in descendants. The parental environment regulates offspring phenotypes potentially via modulating epigenetic marks in the gametes. Here, we review examples of across-generational inheritance of paternal environmental effects and the current understanding of the role of small RNAs in such inheritance. We discuss recent advances in revealing the small RNA payload of sperm and how environmental conditions modulate sperm small RNAs. Further, we discuss the potential mechanism of inheritance of paternal environmental effects by focusing on sperm small RNA-mediated regulation of early embryonic gene expression and its role in influencing offspring phenotypes.

## 1. Introduction

Epigenetic inheritance can be defined as the inheritance of phenotypic changes in the absence of changes in the DNA sequence. In addition to transmitting genetic information in the form of haploid genomes, parents can also transmit epigenetic information to their offspring. A well established example of epigenetic inheritance is genomic imprinting, wherein only one copy of a gene is expressed in a parent-of-origin-specific manner [1]. Moreover, epigenetic marks can be altered by environmental conditions [2]. These observations raise the possibility that exposure to certain environmental conditions can modulate epigenetic marks in gametes (sperm in males and oocytes in females) and influence the phenotypes of offspring. Indeed, there is growing evidence that parental environmental conditions, such as diet, stress, or exposure to toxicants, can influence phenotypes in future generations. Such transmission of epigenetic information from parent to offspring is known as intergenerational inheritance.

It is important to distinguish intergenerational inheritance from transgenerational inheritance [3]. Intergenerational inheritance involves the transmission of epigenetic information from F0 to F1 in the case of males, as F0 sperm that give rise to F1 are directly exposed to the paternal environment. Inheritance of epigenetic information in the F2 and later generations constitutes transgenerational inheritance as those generations are not directly exposed to the paternal environmental insult. In the case of maternal inheritance, as the fetus grows inside the womb, there is a direct interaction of the F1 (fetus) and F2 (fetal germ cells) with the maternal environment. Therefore, inheritance of phenotypic effects in F3 and later generations constitutes transgenerational inheritance. While the mechanism of transgenerational inheritance remains elusive, recent studies shed light on the potential mechanism of intergenerational inheritance. Intergenerational inheritance is mediated by environment-induced changes in the epigenome of the gametes, which in turn affects early embryonic development and offspring phenotype. Most studies on epigenetic inheritance are focused on paternal inheritance, as maternal inheritance remains challenging to unravel mechanistically due to the direct interaction of the growing fetus with the mother’s environment that occludes the ability to decipher consequences of environment-induced molecular changes in the oocyte [4].

The three most well characterized epigenetic information carriers are DNA cytosine methylation, post-translation modification of histones that regulate chromatin states, and small RNAs [3,5]. While a direct role of DNA methylation or histone modifications in intergenerational inheritance remains to be uncovered, an implication of sperm small RNAs in the intergenerational inheritance of paternal environmental effects has been demonstrated by many in the last few years. Here, we will discuss current evidence of intergenerational inheritance via the male germline and mechanisms of sperm small RNA-mediated epigenetic inheritance. We specifically focus on the mechanistic basis of biogenesis of mammalian sperm small RNAs, environmental regulation of levels of small RNAs, and the role of sperm-delivered RNAs in regulating offspring phenotypes. Readers are directed to excellent reviews discussing epigenetic inheritance across other model organisms and the role of DNA methylation and/or histone post-translation modifications in epigenetic inheritance [3,5,6,7].

## 2. Small RNA Classes and Their Functions

A wide variety of distinct species of small RNAs (sRNAs) have been described, including well studied sRNAs, such as microRNAs (miRNAs) and gamete-enriched PIWI-interacting RNAs (piRNAs), as well as relatively understudied sRNAs, such as cleavage products derived from rRNAs and tRNAs [8,9]. Cleavage products of tRNAs are known as tRNA-derived sRNAs or tRNA fragments (tRFs). tRFs are a recently discovered class of sRNAs widespread in most organisms. tRFs were initially thought to be random degradation products of tRNAs. With the advent of sophisticated deep sequencing methods, recent studies revealed that tRFs are generated in a remarkably site-specific manner and are derived from a subset of tRNA isotypes in a given cell type [10]. Fragments of rRNAs (rsRNAs) can be classified into five types based on rRNA precursors (5S, 5.8S, 18S, 28S, and 45S) they are derived from [11]. While it remains unclear if rsRNAs are functional, miRNAs, piRNAs, and tRFs play important roles in gene regulation. sRNAs, such as miRNAs and piRNAs, function by binding to members of a family of effector proteins known as Argonaute (Ago). In complex with Ago, sRNAs regulate gene expression post-transcriptionally by either degrading or deadenylating target RNA or repressing translational [12]. sRNAs can also regulate gene expression by targeting DNA methylation and repressive chromatin formation at the target genes [13,14]. Below, we discuss some of the known functions of sRNAs in regulating spermatogenesis and early embryonic development.

miRNAs are the most well characterized subtype of sRNAs. In the context of reproduction, miRNAs are found in various germ cell populations, including mature sperm [15,16,17], with some miRNAs expressed exclusively in the testes suggesting a role in spermatogenesis [18,19]. Indeed, using Cre drivers for germ-cell-specific deletion of genes involved in miRNA biogenesis or specific miRNA clusters, studies revealed an essential role of miRNAs in germ-cell development and spermatogenesis [20]. Paternal miRNAs and endo-siRNAs are proposed to play an essential role in early embryonic development. Embryos generated using germ-cell specific knockout of Dicer and Drosha, endonuclease enzymes involved in the biogenesis of miRNAs and endo-siRNAs, failed to implant. The implantation defect is potentially caused by disrupted maternal transcript turnover and failure in early zygotic gene activation [21].

piRNAs are 26–31 nt sRNAs highly expressed in germ cells and preferentially bind to the PIWI subfamily of Ago proteins [9,22,23,24,25]. Two major classes of piRNAs have been reported in mammals. The pre-pachytene piRNAs expressed in fetal and newborn mice are homologous to various retroelements and maintain germline genomic integrity by repressing transposon expression [18]. The other class consists of piRNAs enriched during the pachytene stage of meiosis, derived from intergenic regions and proposed to target spermatogenesis-related mRNAs [26]. Mechanistically, piRNAs can regulate transcription by promoting de novo DNA methylation [27] and post-transcriptionally cleaving target transposon mRNA [28]. Male mice lacking PIWI proteins have arrested spermatogenesis and consequently are sterile [29]. Recent work reported that promoter deletion of evolutionarily conserved chromosome 6 piRNA locus 6 (termed *pi6*) affects male fertility [30]. *Pi6* mutant sperm showed capacitation defects and failed to penetrate zona-pellucida, and the embryos generated from *pi6* mutant sperm failed to develop. Mechanistically, *pi6* mutant spermatids showed an increased abundance of mRNAs encoding for proteins involved in sperm acrosome function and zona-pellucida penetration, suggesting a role for piRNAs in regulating mRNAs involved in sperm function [30].

tRFs are the more recently discovered class of sRNAs [31,32,33,34], and are highly abundant in mature sperm [17]. Recent studies shed light on their biogenesis and functions [10,31,35]. sRNAs derived from tRNAs include 28–32 nts 5′ and 3′ halves of tRNAs generated by cleavage in the anticodon loop and 18–22 nts fragments generated via cleavage in the D and T loops of mature or pre-tRNAs [10,36]. One of the earliest discovered classes of tRFs is the stress and starvation-induced tRFs [33,37,38]. Angiogenin, an RNase A family endonuclease, and RNaseT2 endonucleases have been implicated in tRNA processing to generate tRFs [37,38,39,40]. As tRNAs are one of the most highly modified RNA classes, tRNA modifications potentially regulate biogenesis and stability of tRFs [41]. For instance, tRNA methyltransferases Dnmt2 and Nsun2 catalyze tRNA cytosine methylation, which can prevent the cleavage of modified tRNAs by Angiogenin [42,43,44]. Functionally, tRFs play important roles in various cellular processes and are implicated in numerous diseases, including cancer and neurodegenerative disorders [35]. tRFs function by regulating RNA metabolism, translation inhibition [45,46,47], ribosome biogenesis [48], targeted cleavage of 3′ UTRs [49], regulation of apoptosis [50], and regulation of retroviral elements [15,51]. Mature sperm enriched 5′ fragment of tRNA Glycine GCC (tRFGlyGCC) regulates genes involved in mouse zygotic genome activation [15], and a 5′ fragment of tRNA Glutamine TTG (tRFGlnTTG) was reported to participate in the early cleavage of porcine preimplantation embryos [52].

## 3. Current Methods for Sequencing sRNAs

**Figure 1:**
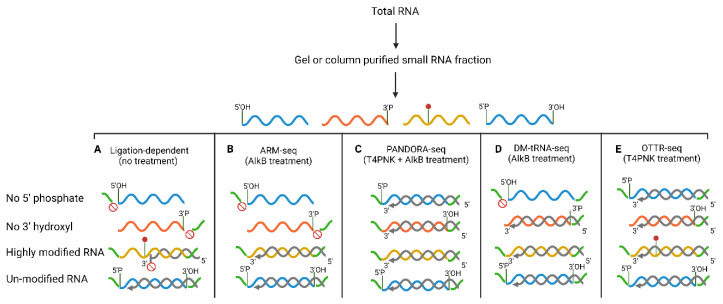
sRNA sequencing approaches: (Different colors represent RNA types harboring specific modification or chemical structures.) (**A**) Ligation-based protocols require 5′ phosphate and 3′ hydroxyl groups for adaptor ligation. In addition, many reverse transcriptase (RT) enzymes cannot read through the hard-stop modifications on highly modified RNA molecules and fall off prematurely. Therefore, small RNAs that are endowed with the above characteristics are not efficiently sequenced. (**B**) To allow sequencing of highly modified RNA molecules, in ARM-seq, RNA is pretreated with AlkB demethylase to remove specific methyl modifications that interfere with RT. (**C**) In addition to using AlkB treatment, PANDORA-seq also uses pretreatment of RNA with T4 Polynucleotide Kinase (PNK) to allow efficient 5′ and 3′ end adaptor ligation. (**D**) DM-tRNA-seq utilizes a combination of AlkB demethylase and a thermostable template-switching reverse transcriptase-TGIRT to overcomes 3′ structure and modification-induced RT stop. (**E**) OTTR utilizes a retroelement reverse transcriptase that allows the addition of 5′ and 3′ adaptors in a single tube during cDNA synthesis by serial template switching.

Next-generation sequencing (NGS) has provided an unprecedented opportunity to discover and quantify diverse classes of sRNA (miRNA, endo-siRNA, piRNA, etc.). However, due to various structural and chemical modifications on sRNAs, depending on the RNA-sequencing protocol used, only a subset of sRNAs are captured in most sequencing libraries [53]. For example, the main steps of a typical sRNA library preparation process include adaptor ligation, reverse transcription, and PCR amplification. All of these steps can introduce bias in the types of sRNA cloned in the sequencing libraries (Figure 1). The ligation-based protocols require 5′ phosphate and 3′ hydroxyl groups for adaptor ligation and are, therefore, most efficient at sequencing miRNAs which naturally have these ends [54]. However, sRNAs such as tRFs and rsRNAs which are generated by endonuclease cleavage (e.g., cleavage of tRNAs by RNase A and T2 family endonucleases) have a 2′, 3′ cyclic phosphate or a 3′ phosphate, which can hinder the capture of these sRNAs in sequencing libraries [55]. Moreover, as tRNAs are highly modified RNA molecules, many reverse transcriptase (RT) enzymes cannot read through the hard-stop modifications on tRNAs and fall off prematurely, synthesizing only partial cDNAs [56]. These partial cDNAs are not amplified during the PCR amplification step as they lack one of the adaptor sequences and remain undetectable in the final sequenced library. All of the above scenarios can drastically influence cDNA synthesis efficacies and exemplify sources of bias in deep sequencing of sRNAs.

Recently developed methods for sRNA sequencing library preparation solved some of the issues mentioned above (Figure 1 and Table 1) and revealed a more comprehensive map of sRNA in various biological samples [8,57,58]. For instance, in ARM-seq and DM-tRNA-seq, RNA is pretreated with an *Escherichia coli* demethylase AlkB to remove N(1)-methyladenosine (m1A), N(3)-methylcytidine (m3C), and N(1)-methylguanosine (m1G) modifications, which are abundant in tRNAs and interfere with RT [58,59]. In addition to using the wild type AlkB, DM-tRNA-seq also uses an engineered mutant (D153S) of AlkB to allow more efficient demethylation of m1G modification [59]. A comparison of sequencing reads from AlkB treated samples to the untreated samples allows identification of AlkB-sensitive methylated nucleotides on a given tRNA. Other sequencing methods use pretreatment of RNA with T4 Polynucleotide Kinase (PNK) to remove 2′, 3′ cyclic phosphates and hydroxylate the 5′ end of the RNA to allow sequencing of previously under-sequenced rsRNAs and some tRFs [8,60].

Additional strategies to overcome structure and modification-induced RT stops, as used in DM-tRNA and mim-tRNA, include using a thermostable template-switching RT such as the thermostable group II intron RT (TGIRT) [59,61]. TGIRT can attach the adaptor to RNA by template switching and thus overcomes the problems with 3′ adaptor ligation bias and tRNA structure [59,61]. Importantly, TGIRT can read through a subset of Watson–Crick face modifications at a reduced fidelity, resulting in misincorporation at modified nucleotides and providing information about potential modified sites in the RNA sequence [59].

A more recently developed method, Ordered Two-Template Relay (OTTR)-seq, uses a modified *B. mori* R2 retroelement reverse transcriptase to fuse 5′ and 3′ adaptors during cDNA synthesis by serial template switching. This streamlined approach obviates the bias introduced during the ligation step. It prevents the inclusion of partial cDNAs into the final library as R2 RT can template jump only when synthesis reaches the 5′ end of an engaged template. In addition to the simplicity and low bias of OTTR-seq, the low input requirement of the protocol also allows better and easier sRNA sequencing of low-RNA-yield samples such as extracellular vesicles and mature sperm [57,62]. PNK treatment can be added to this protocol to further enrich sRNA classes, as discussed above [62,63].

Direct RNA sequencing and machine learning have also been applied to sequence sRNAs. Oxford Nanopore Technologies (ONT), a single-molecule long-read sequencing method, has been used to sequence and map modifications on tRNAs [64]. Nanopore sequencing of tRNAs and other sRNAs has many technical advantages. It does not require bias-inducing steps such as RT and amplification and allows the detection of modified nucleotides as a part of sequencing. Importantly, due to long-read sequencing, Nanopore sequencing can detect multiple modifications on a single RNA molecule. This method has been successfully applied to sequence all 43 expected isoacceptors in *E. coli* [64]. While Nanopore technology has great promise, it is still in its infancy concerning sequencing more complex and highly modified mammalian tRNAs and shorter RNAs such as tRFs.

## 4. The Mammalian Sperm RNA Payload

**Figure 2:**
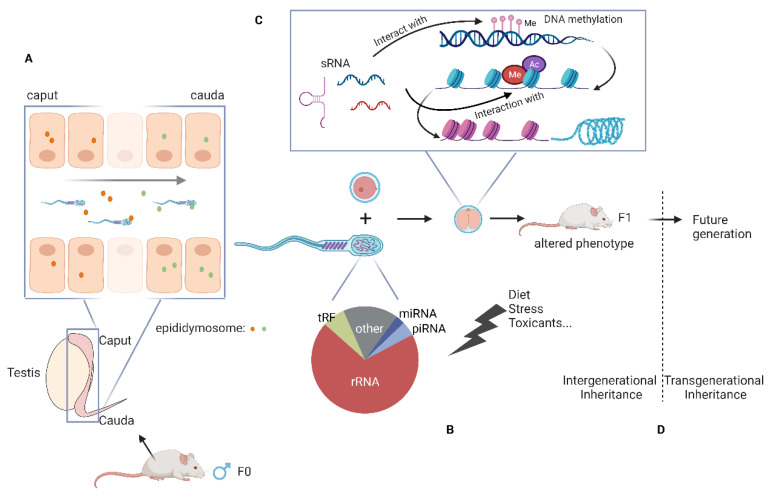
Model for intergenerational inheritance via sperm RNAs: (**A**) Epididymis shapes the small RNA payload of mature sperm. Small RNAs are delivered from the epididymis epithelial cells to sperm via extracellular vesicles known as epididymosomes represented by orange and green dots to depict different types of vesicles. (**B**) Mature sperm in mice are chiefly comprised of rsRNAs, tRFs, miRNAs, and piRNAs. Various studies demonstrate that levels of sperm small RNAs are altered in response to paternal environmental conditions. (**C**) Different epigenetic marks, including DNA methylation, histone modifications, and small RNAs, can function synergistically to regulate intergenerational epigenetic inheritance and influence offspring phenotypes. (**D**) In males, intergenerational inheritance involves the transmission of paternal environmental effects from F0 to F1. In some instances, paternal environmental effects can be transmitted to F2 and later generations, constituting transgenerational inheritance.

With constantly improving sRNA sequencing methods (as discussed above), we now have a more comprehensive picture of the sRNA payload of mature sperm. The mouse sperm chiefly consist of rsRNAs, tRFs, miRNAs, and piRNAs, with rsRNAs and tRFs comprising more than 80% of the sequencing reads [8,60] (Figure 2). In human sperm, rsRNAs derived from 28S rRNA are most abundant, accounting for ~60% of the total rsRNAs [65]. In addition to sRNAs, other non-coding RNAs identified in human and mouse testicular spermatozoa and sperm are circular RNAs (circRNAs). CircRNAs have been proposed to function as miRNA sponges, protein scaffolds, and translation templates [66,67]. More recently, the long RNA (>200 bp) profile of sperm has been revealed by Isoform Sequencing (Iso-seq) using PacBio SMRT (Single Molecule, Real-Time) technology [68]. This study identified >3000 full-length intact long RNA transcripts in sperm of mice and humans, distinct from the long RNA profile of testes [68]. The sperm-specific long RNAs consisted of both mRNAs and long-noncoding RNAs. Interestingly, intact mRNAs enriched in sperm encode for ribosomes, suggesting a role for these transcripts in regulating early embryonic protein synthesis.

### The Epididymis Shapes the sRNA Payload of Mature Sperm

Small RNA sequencing of male germ cells at different stages of development revealed that sperm undergo dynamic changes in their sRNA payload [15,60]. Spermatogenesis occurs in the testis, following which the testicular spermatozoa enter a long-convoluted tubule known as the epididymis where sperm mature and acquire motility and fertility [69]. Sperm sRNA payload is dramatically altered during sperm epididymal maturation. While testicular germ cells are highly abundant in piRNAs, the mature epididymal sperm are chiefly comprised of tRFs, miRNAs, and rsRNAs [8,60].

Sperm entering the epididymis are transcriptionally silent, raising the question of how sperm acquire new sRNAs during maturation in the epididymis. Studies in various model organisms, including worms, flies, and plants, suggest that RNAs produced in somatic support cells are transferred into developing germ cells [70,71]. Several lines of evidence support that RNA shipment from the somatic cells of the epididymis shapes the sRNA payload of mammalian sperm [72,73] and that this soma-germline communication is potentially mediated via extracellular vesicles (EVs) secreted from the epididymis epithelium cells known as epididymosomes [60,72,74] (Figure 2). First, the sRNA payload of sperm matches well with the sRNA profile of epididymosomes [15,74,75,76]. Second, in vitro assays revealed that incubation of immature sperm from the testis or caput epididymis (proximal epididymis) with epididymosomes from caput or cauda epididymis (distal epididymis, where mature sperm are stored) resulted in an sRNA profile of “reconstituted” sperm that matches well with that of caput or cauda sperm, respectively [15,60,74,77]. Third, miRNAs metabolically labeled in the epididymis were detected in mature sperm, providing in vivo evidence that at least a subset of sRNAs in mature sperm are synthesized in the somatic cells of the epididymis [60]. Finally, reconstitution of naïve sperm sRNA payload by incubating with epididymosomes purified from males exposed to environmental stressors resulted in the transmission of paternal environmental effects to offspring generated from the reconstituted sperm [78,79]. While there is strong evidence for epididymis-delivered sRNAs in shaping the mature sperm RNA payload, loss of testis-specific RNAs, such as piRNA, and/or in situ generation of sRNAs from precursor RNAs likely also contribute to the altered levels of sRNAs in sperm during epididymal maturation.

## 5. Paternal Environmental Effects on Offspring Phenotypes

Some of the earliest hints on paternal epigenetic inheritance came from human epidemiological studies, which suggested that ancestral diet can influence metabolic phenotypes in descendants. Grand parental food availability was linked to mortality rates in grand offspring [80,81,82]. Interestingly, the effects observed were sex-specific. For example, the grandfather’s diet affected his grandsons, and the grand-maternal diet influenced the granddaughter’s metabolic health. This study suggests a sex chromosome-specific function in such inheritance; however, the underlying mechanism of this sex-specific effect remains unclear. Similarly, epidemiological studies of the Dutch Hunger Winter of 1944–1945 indicated that maternal starvation during pregnancy could affect metabolic health in adult offspring [83].

In the past decade, numerous rodent model studies have been conducted to investigate the phenomenon, and these studies demonstrated that paternal exposure to different environmental conditions could influence offspring phenotypes. To study intergenerational inheritance of paternal environmental effects, males are exposed to a specific environmental perturbation either in utero, from weaning to adulthood, or during adulthood, and then mated with control females. In some studies, in vitro fertilization (IVF) or intra-cytoplasmic sperm injections (ICSIs) are performed to generate zygotes that are implanted in surrogate females. As parents only contribute their gametes in assisted fertilization procedures, the latter approaches allow the assessment of transmission of environmental information exclusively via gametes. Next, phenotypic changes are examined in the offspring of exposed males compared to phenotypes in the offspring of unexposed males. A wide range of paternal exposures has been studied so far. The major classes of exposures studied include dietary alterations such as high fat, low protein, or Western diet [16,84,85,86,87,88,89,90,91], exposure to drugs such as nicotine and ethanol [92,93,94,95,96,97], paternal obesity [87], fear-conditioning [98], endocrine disruptors [99,100], early life trauma and chronic stress [101,102,103,104,105,106,107], temperature [108,109,110], and dietary supplementation with folate [111] and vitamins [112,113].

It is generally observed that the paternal environment can influence metabolic and behavioral phenotypes in offspring. For example, exposure of F0 male mice to a high-fat diet (HFD) led to diet-induced obesity in F1 female offspring and insulin resistance in offspring of both sexes [84,91]. Similarly, F1 offspring of males fed a low-protein diet exhibited elevated hepatic gene expression of lipid and cholesterol biosynthesis and decreased levels of cholesterol esters [85]. Furthermore, diet can impact phenotypes across multiple generations. Paternal F0 HFD alters the metabolic health of second-generation offspring in a sex-specific parental lineage [87]. In addition, numerous studies in mice have used interventions such as preconception fasting and fetal undernutrition to establish a link between paternal nutrition and the metabolic health of offspring [88,90,114]. Recent studies also report that paternal exercise can suppress the transmission of high-fat diet-induced metabolic defects in offspring [115]. Paternal exposure to psychological stress typically leads to reduced stress sensitivity and altered cortisol levels. Interestingly, offspring of stress-exposed males also show metabolic changes, including altered glucose metabolism [101,102,103,104,105,106,107]. Paternal nicotine exposure has been reported to affect offspring metabolism and behavior [93,97]. In some cases, the reproductive system and fertility are also affected. For example, F1 male offspring of Vinclozolin-exposed mothers displayed infertility. This defect was transmitted to males until the F4 generation [100], suggesting that paternal environmental conditions can influence the health of offspring across multiple generations. It is important to note that epigenetic alteration can also promote genome instability, leading to an accelerated accumulation of genetic mutations, which could play a role in stabilizing the trait in multiple generations [116].

Further, low protein diet fed males have been reported to sire offspring with reduced male-to-female ratio and increased body weight in males. In addition, offspring of both sexes exhibited vascular dysfunction and lowered glucose tolerance, whereas only the male offspring had mild hypotension and elevation of heart rate [88]. Paternal low protein diet is also associated with increased fetal:placental weight ratio and perturbed fetal skeletal development [117]. Recent studies also provide evidence that in utero environmental exposure to specific diets or drugs can induce cancer predisposition in offspring [118,119,120]. Paternal low protein diet is associated with increased breast cancer risk in offspring [121]. Male mice were fed a low protein diet or control diet, and their female offspring were exposed to dimethylbenz[a]anthracene (DMBA) to induce mammary carcinogenesis. Female offspring of male mice fed a low-protein diet have higher incidence of mammary tumors compared to offspring of control male [121]. Diet-induced paternal overweight is also linked to increased risk of breast cancer in offspring [122].

While the association between paternal diet, offspring growth and adult disease risk is well characterized, the effect of a father’s diet may start as early as post-fertilization. Paternal obesity led to significant delays in cell cycle progression during preimplantation embryo development, leading to reducing blastocyst cell numbers. Glucose consumption is also increased in embryos derived from obese fathers [123]. It is noteworthy that in some instances, offspring of exposed males display a phenotypic effect compared to naïve offspring only when challenged with the same or a related stressor. For example, in a 6-week paternal chronic variable stress exposure, F1 offspring of exposed males display similar immobility time and sucrose intake as the control mice. However, when subjected to chronic variable stress for two consecutive weeks, the offspring of exposed males recapitulated the paternal depressive-like phenotypes [105]. These observations suggest that while an environment-induced molecular change is inherited by offspring (for instance, sperm RNA changes as discussed below), a phenotypic manifestation of this change could be induced by exposure of the offspring to a similar environmental challenge. These studies further reinforce the necessity of comprehensive molecular and phenotypic assessment in multigenerational studies to capture the effects of the paternal environment fully.

## 6. Role of Sperm Non-Coding RNAs in Intergenerational Inheritance

Given that mature sperm carry little cytoplasm, and most long RNAs in sperm are degraded during spermatogenesis, it is debated whether sperm sRNAs are delivered to the embryo at the time of fertilization and whether they are functional in the embryo. Some of the earliest studies by the Krewatz lab on sperm RNAs reported that human sperm carry RNAs that can be detected in fertilized embryos [124]. A more direct role of RNA in mammalian epigenetic inheritance was demonstrated by paramutation in mice [125]. Paramutation is a phenomenon first reported in plants [126] and involves interallelic interactions wherein one allele (paramutagenic allele) causes heritable changes in the expression state of the other allele (paramutable allele) [127]. The paramutable allele acquires paramutagenic capacity in the following generations. Studies in plants implicate a role of sRNAs in trans-interaction between the two alleles [128]. The *Kit* gene in mice encodes a receptor tyrosine kinase. A null mutant allele (*Kit^tm1Alf^*) is lethal in the homozygous state, and the viable heterozygous animals (*Kit^tm1Alf^*^/+^) have white tail tips and feet. When heterozygous animals (*Kit^tm1Alf^*^/+^) were mated, most of the genotypically wild-type progeny had white patches characteristic of paramutagenic allele (referred to as Kit* phenotype). These studies suggested that the paramutagenic allele (*Kit^tm1Alf^*) could silence the wild-type allele [125]. Mechanistically, Kit* mice showed reduced levels of polyadenylated mature *Kit* mRNA. DNA cytosine and histone methylation were not altered at the *Kit* promoter region; instead, there was an accumulation of shorter fragments of *Kit* RNA in various tissues, including sperm. Interestingly, microinjection of total RNA isolated from either brain or sperm of *Kit^tm1Alf^*^/+^ heterozygote mouse into naïve wild-type zygotes generated progeny with white patches. It is important to note that microinjections of RNA isolated from WT phenotype animals also resulted in white patches in progeny, albeit at a significantly lower frequency than those observed after microinjection of RNA purified from *Kit^tm1Alf^*^/+^ heterozygote mouse tissues. Finally, microinjection of miRNAs mir-221 and mir-222, which target *Kit* mRNA, in naïve zygotes resulted in white patch phenotype [125], suggesting that miRNA-mediated regulation of *Kit* mRNA in the early embryo is sufficient to induce a heritable change in gene expression.

The role of sperm RNAs in intergenerational inheritance has become more evident in recent years. Various studies in rodents demonstrate that levels of sperm sRNAs, including tRFs, rsRNAs, miRNAs, and piRNAs, are altered in response to environmental conditions such as malnutrition and paternal obesity [15,16,87,129], exposure to toxicants, such as vinclozolin, DDT, ethanol, and nicotine [104,130,131], and psychological stress, such as early life trauma or chronic stress [101,105,132,133,134]. Changes in specific sRNA levels were also observed in mice exposed to long-term (12 weeks) [135] and short-term (4 weeks) [136] wheel-running. While many studies demonstrate changes in sRNA levels in sperm of exposed males, a direct causal role of those RNAs in transmitting paternal environmental effects to offspring is explored in only a few studies. Below, we describe some of those studies in more detail (Table 2) and the potential mechanisms by which sperm RNA payload can transmit paternal environmental information to offspring (Figure 2).

### 6.1. tRNA Fragments

The role of tRFs in intergenerational inheritance was first discovered in two dietary intervention studies in mice [15,16]. Male mice fed a low protein diet sired offspring with altered hepatic lipid and cholesterol biosynthesis [85]. Sperm of mice on a low protein diet displayed increased levels of many tRFs, including tRFGlyGCC. Microinjection of a mimic of tRFGlyGCC into naïve embryos resulted in transcriptional repression of genes driven by the LTR of an endogenous retroviral element (MERVL) [15]. Mechanistically, tRFGlyGCC regulates the biogenesis of noncoding RNAs involved in histone pre-mRNA processing, and thus, affects the levels of histone proteins and chromatic compaction at MERVL genes [137]. Given that MERVL genes are known to regulate zygotic genome activation and constitute the totipotency program of the embryo [138], these studies suggest that diet-responsive tRFGlyGCC potentially regulates cell fate allocation and placental function, which in turn can regulate metabolic health in adulthood.

In a study examining the inheritance of effects of a paternal high-fat diet, sperm heads from male mice fed a high-fat diet were injected into control oocytes to produce offspring [16]. ICSI was used to directly test epigenetic inheritance via sperm and eliminate other confounding factors, for example, seminal fluid, microbiome, etc. The progeny of high-fat diet (HFD) mice showed impaired glucose tolerance and insulin resistance. Sperm of HFD mice showed an overall increase in the levels of tRFs. Intriguingly, embryos injected with 30–40 nts RNAs (corresponding to tRF size) purified from high-fat sperm produced offspring with altered glucose tolerance, suggesting that tRFs (and other sperm RNAs of 30–40 nts size range) are responsible for the transmittance of acquired metabolic disorder to the next generation [16].

tRFs are derived from the most highly modified RNA species—tRNA. Levels of two tRNA modifications, 5-methylcytosine (m5C) and N-2-methylguanosine (m2G), increased in 30–40 nts fraction of RNAs purified from HFD sperm, suggesting that exposure to HFD resulted in higher levels of these two modifications in tRFs. Consistently, microinjection of tRFs isolated from HFD sperm, not synthetic tRF mimics, recapitulated the glucose-intolerance phenotype in the offspring resulting from injected embryos [16]. These observations are consistent with a previous study showing that RNA-modifying enzyme DNA (cytosine-5)-methyltransferase-like protein 2 (Dnmt2) is critical for the inheritance of epigenetic variations [139]. More recently, it was reported that Dnmt2 mediates intergenerational inheritance of paternally acquired metabolic disorder potentially by regulating sperm tRF and rsRNA levels and modifications. Offspring produced by microinjection of RNAs purified from *Dnmt2*^−/−^ deletion HFD mice did not show metabolic phenotypes displayed by offspring of wild-type mice on HFD [140]. Dnmt2 is responsible for adding m5C to several types of tRNAs, and along with another related protein, Nsun2, it is important for maintaining tRNA stability and preventing tRNA cleavage by Angiogenin [42,44]. There was a significant decrease in the levels of m5C in 30–40 nts RNA purified from sperm of *Dnmt2*^−/−^ mice [16], suggesting that Dnmt2-mediated m5C modification regulates tRF levels in sperm and their function in the intergenerational inheritance of metabolic phenotypes. These findings are, however, inconsistent with the observation that loss of Dnmt2 resulted in a decrease in tRFs and needs further exploration. Another study reported that mice exposed to ethanol have higher levels of 5′-methylaminomethyl-2-thiouridine (mnm^5^s^2^U) and formylcytidine (f^5^C) in sperm sRNA fraction [104], suggesting that ethanol exposure affects specific modifications of tRFs.

In addition to the RNA sequence, RNA modifications have emerged as a new player in the transmission of epigenetic memory of paternal exposures. RNA modifications can stabilize the molecule, thus allowing a longer half-life for RNA species to exert their cellular functions. Moreover, certain modifications can facilitate interactions with RNA-binding proteins, which can further stabilize the RNA molecules and cooperate in their functional output. Given the high levels of modified nucleotides in tRNAs (more than 100 known modifications), epigenetic inheritance by tRFs is potentially regulated by RNA modifications. Further development of methods to detect and quantify RNA modifications is required to gain insights into the role of additional RNA modifications in epigenetic inheritance.

### 6.2. Micro-RNAs

Numerous paternal exposure studies report miRNA changes in sperm of exposed mice [141]. For example, the abundance of let7c miRNA changed in sperm in response to a high fat [129] and a low protein diet [15]. Rats fed a high-fat diet for 12 weeks sired offspring with reduced body weight and increased glucose tolerance when fed a control diet. Let7c miRNA was upregulated, and mir-293-5p and miR-880-3p were downregulated in sperm of high-fat diet fed fathers (F0-HFD) and their sons fed a control diet (F1-CD). Mating these F1-CD males with females on a control diet produced F2 females with a similar decrease in body weight and increased glucose tolerance, suggesting that the F1 sperm could potentially transmit F0-HFD effects to the F2 generation. Let7c was also differentially expressed in the liver, and adipose tissues of high-fat diet fed F1 and F2 offspring of F0-HFD fathers [129]. These studies propose that changes in Let7c in sperm of F0 could lead to changes in Let7c in offspring tissues, including sperm, and transmit paternal high-fat effects to future generations. However, both high-fat and low-protein diets affect offspring metabolism, but Let7c levels are downregulated in low-protein sperm [15]. Moreover, although Let7c is known to regulate metabolism [142], a direct link between metabolic phenotypes in offspring and sperm-delivered Let7c miRNA remains to be established.

Interestingly, a high-fat diet can elicit opposing effects based on the composition of the fat [143]. A paternal lard-based high-fat diet which is rich in saturated fatty acids increases the risk of breast cancer in female offspring compared to plant-based high-fat diet or control diet. These effects are potentially linked to alterations in miRNA expression in fathers’ sperm and their daughters’ mammary glands. Paternal low protein diet can modulate the birthweight and later breast cancer risk of daughters. This phenotype is associated with differential expression of sRNAs including tRF and miRNA which regulate AMPK energy-sensing pathway in mammary glands [121].

**Table 2:** Overview of the different classes of sRNAs altered in sperm of animals exposed to various environmental conditions. NA: Information about sRNA sequencing method not available.

Environmental Factors	Tissue (Species)	Altered sRNA Subtype	Primary Detection Method	Citation
High-fat diet	F0 Sperm (rat)	15 miRNAs (let-7c, miR-293, miR-880, etc.), 41 tRFs (tRFGluCTC etc.) and 1092 piRNAs (piR-025883, piR-015935, piR-036085)	NEBNext^®^ Multiplex Small RNA Library Prep Set for Illumina (New England Biolabs, Ipswich, MA, USA)	[129]
High-fat diet	F0 Sperm (mouse)	11.53% tRFs (tRFGluCTC, tRFGlyGCC etc.), and 2.28% miRNAs (miR-3107, miR-142, miR-7210, etc.)	TruSeq Small RNA Sample Prep Kit (Illumina, San Diego, CA, USA)	[16]
High-sugar diet	F0 Sperm (mouse)	miR-19	-	[86]
Western diet		RsRNAs (16S, 18S, 28S), tRFs (tRFSerGCT, tRFGluCTC, tRFiMetCAT, miRNAs (miR-10b, miR-10a, miR-125a)	TruSeq Small RNA Sample Pre Kit (Illumina)	[144]
Low-protein diet	F0 Sperm (rat)	tRFGlyGCC, tRFGlyCCC, tRFLys CTT, tRFHisGTG and miRNA Let7	3′ and 5′ ligation-dependent protocol	[15]
vinclozolin	F3 Sperm (rat)	13 miRNAs (miR-101, miR-1247 etc.), 16 piRNAs (piR_000216, piR_000333 etc.), 19 Mitochondrial RNAs, 59 tRFs (tRFProAGG, tRF AlaAGC etc.)	Ion RNA Kit v2 (Life Technologies, Carslbad, CA, USA)	[130]
DDT	F1, F2, and F3, sperm (rat)	tRFs and piRNAs	NEBNext Multiplex Small RNA Library Prep Set for Illumina	[131]
chronic mild stress (CMS)	F0 Sperm (mouse)	miRNAs (miR-199a, miR-103, miR-146a etc.)	TruSeq Small RNA Sample Pre Kit (Illumina)	[105]
chronic variable stress (CVS)	F0 Sperm (mouse)	miR-29c, miR-30a, miR-30a, miR-32, miR-193-5p, miR-204, miR-375, miR-532-3p, miR-698	qRT-PCR	[101]
chronic social instability (CSI)	F0 Sperm (mouse)	miR-449, miR-34	qRT-PCR	[145]
unpredictable maternal separation combined with unpredictable maternal stress (MSUS)	F1 Sperm (mouse)	miRNAs (miR-375-3p, miR-375-5p, miR-200b-3p etc.)	modified Illumina v1.5 (3′ and 5′ ligation-dependent protocol)	[133]
hypothalamic–pituitary–adrenal (HPA) axis dysregulation by feeding corticosterone (CORT) for 4 weeks	F1 Sperm (mouse)	miRNAs (miR-144, miR-190B, miR-192 etc.)	NA	[134]
long-term exercise	F0 Sperm (mouse)	miR-483, miR-431, miR-221, miR-21	qRT-PCR	[135]
short-term exercise	F0 Sperm (mouse)	miR-19b, miR-455 and miR-133a, tRFGly and tRF-Pro	NA	[136]

A few recent studies explored the causal link between miRNA changes in sperm and altered phenotypes in offspring. A paternal high-sugar and high-fat diet (Western-like diet, WD) led to increased body weight and impaired glucose tolerance and insulin resistance [86]. Microinjection of purified RNAs from the testis and sperm of WD mice into naïve embryos resulted in altered metabolic phenotypes in the offspring, suggesting a role of RNA in this inheritance. Analyzing sRNA levels in the testis and sperm of F0 WD males revealed differential expression of various sRNAs, including miR19b. Microinjection of miR19b into a naïve embryo produced offspring (R1-miR19b) with increased weight, and a subset also showed impaired glucose tolerance. Intriguingly, when R1-miR19b males were mated with control females, their offspring (R2-miR19b) also displayed metabolic phenotypes, although the penetrance of this phenotype was variable across individuals [86]. A more recent study from the same group demonstrated that exposure to WD for five consecutive generations resulted in a progressive dysregulation of metabolic phenotypes in males [144]. There was an exacerbated weight gain over generations. The weight gain was associated with increased perigonadal white adipose tissue, and mice exposed to WD for five generations (WD5) also displayed a fatty liver phenotype. Consistent with the transgenerational inheritance of WD metabolic phenotype, the offspring generated from WD5 showed increased weight for at least four generations. Notably, weight gain was not associated with increased glucose metabolism in WD5 offspring. Microinjection of total sperm RNA from WD5 males into naïve embryos transmitted metabolic phenotypes only until F2 generation [144], suggesting that sperm RNAs are not sufficient to transmit paternal environmental information transgenerationally. Other epigenetic mechanisms potentially play a role in the transgenerational transmission of paternal diet-induced obesity effects (discussed later).

Psychological stress also changes miRNA levels [105,132,145]. One such paradigm is chronic variable stress (CVS), wherein mice are exposed to variable stressors (novel object, restraint, loud noise, predator odor, etc.) once per day for a total of 6 weeks post-weaning. When mated with control females, CVS males sired offspring with reduced hypothalamic–pituitary–adrenal (HPA) stress axis responsivity and altered gene expression in the stress-regulating regions of the brain. Sperm of CVS mice showed increased levels of nine miRNAs, namely miR29c, miR30a, miR30c, miR32, miR193-5p, miR204, miR375, miR532-3p, and miR698 [101]. Remarkably, microinjection of all nine miRNAs upregulated in CVS sperm into control zygotes, but not single miRNA injections, produced offspring with reduced HPA stress axis [132], demonstrating a causal role of nine miRNAs in regulating offspring stress-responsivity phenotypes. Mechanistically, injecting microRNAs in early zygotes downregulated most of the target mRNAs examined, consistent with the hypothesis that sperm-delivered miRNAs target the maternal store of mRNA transcripts [132]. Further, the fusion of control sperm with epididymosomes isolated from stress hormone-treated epididymis epithelial cells led to a stress-like miRNA profile of “reconstituted” sperm. Embryos generated using those reconstituted sperm produced progeny with stress-exposure phenotypes, suggesting that miRNAs that change in sperm in response to stress are generated in the epididymis and shipped to sperm via epididymosomes [79] (Figure 2). While these studies provide compelling evidence for the role of sperm sRNAs in regulating offspring health, it remains unknown how transcriptome alterations in early preimplantation embryo impact gene expression in specific brain regions and adult behavior.

Using a chronic mild stress (CMS)-induced depression-like mouse model, another research team reported the role of miRNAs in the transmission of paternal stress effects [105]. Mice exposed to CMS developed depressive-like phenotypes such as immobility and increased blood corticosterone levels. CMS offspring also displayed a depressive-like phenotype after being challenged with a short (2 weeks) chronic variable stress compared to control animals. A group of 16 miRNAs was significantly upregulated in sperm of mice exposed to CMS. The authors were able to recapitulate the depressive-like phenotype in the F1 offspring produced from embryos injected with 16 synthetic stress-responsive miRNAs. Further, interfering with the function of these specific miRNAs (by injecting antisense oligos) in embryos generated using CMS sperm rescued the depressive-like phenotype and produced normal offspring [105]. Exposure to corticosterone stress hormone was also shown to change sperm miRNA levels and phenotypes in F1 and F2 offspring, suggesting that glucocorticoid signaling facilitates transmission of paternal stress-exposure phenotypes transgenerationally [134].

To examine whether human sperm similarly respond to stress as mouse sperm, sperm sRNA payload of individuals exposed to early life stress (based on responses to the Adverse Childhood Experience questionnaire) and mice exposed to adolescent chronic social instability (CSI) stress was examined. Multiple miRNAs of the miR339/34 family were downregulated in sperm of mice and men subjected to early life stress. Interestingly, the changes in miR449/34 levels persisted in embryos generated using sperm from mice exposed to stress through at least the morula stage and in the sperm of F1 offspring, suggesting that these miRNA changes potentially contribute to the transmission of stress phenotypes across generations [145]. However, in the absence of a known mechanism to amplify miRNAs in mammals, how sperm delivered miRNAs are amplified and maintained during early embryonic cell divisions remains unknown.

### 6.3. PIWI-Interacting RNAs

piRNAs are known to regulate multigenerational epigenetic memory in *C. elegans* [146,147]. Rodent studies also implicated piRNAs in the intergenerational inheritance of paternal environmental effects. In mice, unpredictable maternal separation combined with unpredictable maternal stress (MSUS) resulted in behavioral deficiencies in adulthood and transmission of those effects to offspring [102]. Deep sequencing of sRNAs from sperm of MSUS mice revealed changes in the abundance of sRNAs, including piRNAs from cluster 110 on chromosome 13 [133]. Microinjection of RNA purified from MSUS sperm in naïve zygotes produced progeny with altered behavior and metabolism, demonstrating a causal role of sperm RNAs in the transmission of MSUS effects. However, the identity of the specific RNA(s) involved in such inheritance remains unknown.

In the study using the Western-like diet discussed above, in addition to miRNAs, 190 piRNAs spread across 63 clusters in the testis of exposed males were also differentially expressed [86]. In rats, chronic exposure to a high-fat diet resulted in altered body weight and glucose levels in F1 and F2 offspring. At the molecular level, F0 sperm displayed changed levels of 1092 piRNAs [129]. To determine whether specific sRNAs are responsible for transgenerational transmission of paternal dietary effects, common sRNAs altered in sperm of F0 males fed a high-fat diet and their F1 offspring maintained on standard chow were examined. Three piRNAs (piR-025883, piR-015935, piR-036085) and miRNA Let7c changed similarly in the sperm of F0 fathers fed a high-fat diet and their sons [129]. Changes in levels of piRNAs were also reported in sperm of the F3 generation of mice exposed to vinclozolin and DTT [130,131]. However, none of the above studies investigated the functional consequences of changes in piRNA levels in the testis and sperm. Future studies are needed to determine whether piRNAs can serve as molecular carriers of paternal environmental effects.

### 6.4. Circular RNAs

Circular RNAs (circRNAs) are generated by a back-splicing reaction that covalently binds a downstream splice donor site reversely with an upstream splice acceptor site. Many protein-coding genes can generate circRNAs through the back-splicing of their exons. Sequencing of non-polyadenylated transcriptome uncovered the presence of circRNAs in numerous organisms, including protozoa, fungi, plants, and animals [148]. CircRNAs have been recently identified in human, pig, and mouse sperm and function as miRNA sponges, protein scaffolds, and translation templates [66,67,149,150]. Thus, circRNAs are interesting candidates for epigenetic germline inheritance. The functional characterization of circRNAs in paternal epigenetic inheritance is still in its infancy, but recent studies shed some light on the role of circRNAs in epigenetic inheritance. Activation of the stress-sensitive glucocorticoid receptor (GR) in mice results in an altered RNA payload of F0 sperm, changes in early embryonic transcriptome, and offspring metabolism. Two specific circRNAs were upregulated in sperm of GR mice, and one of the mRNA targets of their miRNA-sponge was increased in embryos, which agrees with the rationale that dysregulated circRNA levels impact their ability to bind and inhibit their miRNA target, thus impeding the miRNA inhibitory functions in gene regulation [151]. In another study, incubation of sperm with epididymosomes resulted in the uptake of CNOT6L mRNA and FUS protein by sperm and generation of circRNA circCNOT6L in sperm, which in turn was delivered to the oocyte to regulate maternal mRNA stability [152]. These studies warrant further investigations into whether circRNAs serve as carriers of paternal environmental information across generations.

### 6.5. Long RNAs

In addition to sRNAs, long RNAs in sperm may also play a role in intergenerational inheritance [153]. In the mice model of MSUS described above, when naïve embryos were injected with long RNA (>200 bp) or sRNA (<200 bp) fractions from MSUS sperm, the offspring recapitulated different hallmarks of the MSUS phenotype. Given that total RNA-injected embryos can develop behavioral alterations observed in offspring generated by natural mating, it is suggested that both sRNAs and long RNAs harbor some stress-responsive changes that separately confer behavioral phenotypes to offspring. Further investigations into sperm long RNA changes in response to environmental conditions by long-read sequencing, such as ONT, will provide insights into their identity and role in intergenerational inheritance.

## 7. Interplay among Different Epigenetic Markers

Mammals do not express an RNA-dependent RNA polymerase (RdRP) that is known to amplify sRNAs in plants and worms [154,155]. Therefore, in the absence of a known mechanism for amplifying sperm-delivered sRNAs in the embryo, it is unclear how sRNA-mediated changes in early preimplantation embryos persist throughout development to give rise to a phenotype in adult offspring. One possibility is that sperm sRNAs could initiate a cascade of transcriptional regulatory events, whereby products of genes regulated by sRNAs in early embryos could regulate later stages of development. For example, sperm sRNAs could regulate the expression of some key transcription factors at early embryonic stages, and those transcription factors could regulate gene expression at later stages of development.

It is worth noting that studies that uncovered the potential role of sRNAs in paternal epigenetic inheritance do not rule out the possibility that sRNAs and other epigenetic regulators, such as histone modifications and DNA methylation, function synergistically to regulate intergenerational epigenetic inheritance. sRNA-mediated control of DNA methylation and histone modifications facilitates transgenerational inheritance in plants and worms [3,7,156,157]. Similarly, mammalian paternal sRNAs could alter histone modifications or DNA methylation in early embryos [137] (Figure 2). In that way, an acute alteration in sRNAs in the early embryo in response to the paternal environment can induce a more stable form of epigenetic change in gene expression that can be faithfully copied as the embryo grows and thus lead to paternally acquired phenotypes in adult offspring. Consistent with this hypothesis, various non-coding RNA species, including miRNAs and long non-coding RNAs, are critical for regulating DNA methylation via either controlling DNA methyltransferases expression or directly interacting with DNA methyltransferases at specific genome loci [158,159]. Another key protein that regulates single-stranded RNAs and local chromatin structure is Argonaute (Ago), a member of a highly evolutionarily conserved family of RNA-binding proteins. For example, the miRNA-Ago complex target chromosome sites in trans and recruit additional chromatin-modifying factors to affect local chromatin structure, leading to changes in gene transcription or altered alternative splicing events [160].

## 8. Role of Sperm sRNAs in Intergenerational Inheritance: Evidence from Human Studies?

Although limited, there is some evidence of sperm sRNA changes in response to environmental conditions in humans, including studies on early life trauma [145], exercise [161], high sugar [162], and obesity [163]. As discussed above, in men with a history of childhood trauma, miR-449 and miR-34 were altered in their sperm. In the mouse model of early life stress, those changes in sperm miRNA content are also persistent in early embryonic development and unexposed F1 offspring of the stressed father [145]. In a study evaluating the impact of smoking cigarettes, around 30 miRNAs were significantly altered in the spermatozoa of smokers compared with non-smokers. These altered miRNAs appear to mediate pathways vital for healthy sperm and normal embryo development [164]. In another epidemiological study of paternal obesity, sRNA abundance and DNA methylation patterns were markedly different in lean and obese men. Moreover, surgery-induced weight loss was associated with a dramatic remodeling of sperm DNA methylation at genetic loci that control appetite [163]. An examination of the impact of exercise on human sperm showed changes in piRNAs after six weeks of endurance training. These changes were reverted after the cessation of training for three months [161].

While there is evidence of changes in sperm sRNA levels in sperm of men exposed to various environmental stressors, a link between paternal exposure-induced alteration in sperm RNAs and specific phenotypes in offspring is still lacking due to several obvious reasons. Tracking human exposure and evaluating offspring traits are particularly difficult given the long lifespan of humans. Further, the dynamic environment an individual is exposed to during their lifespan adds many confounding factors and makes data interpretation more challenging. Studies combining human epidemiological data and experimental results from mammalian models will be critical to understanding the conserved mechanisms of paternal epigenetic inheritance.

## 9. Conclusions and Future Directions

There is growing evidence that the paternal environment can influence phenotypes in future generations. Recent studies implicate sperm sRNAs in such inheritance. Numerous studies provide evidence that paternal environmental conditions, such as altered diet, stress, or toxicant exposure, can alter levels of specific sRNAs in sperm. Some recent studies demonstrated that microinjection of sperm sRNAs, purified from sperm of exposed males or environmentally-responsive synthetic sRNAs, in naïve embryos produced offspring with complete or partial phenotypic effects of paternal environmental exposure. These observations suggest that sperm sRNAs have a causal role in transmitting paternal environmental information to offspring.

While sRNAs have emerged as a key player in the intergenerational epigenetic inheritance of paternal environmental effects, many questions remain unanswered regarding their role in regulating offspring phenotypes. First, sperm RNA content is minimal compared to the maternal RNAs stored in the oocyte [165]. For example, some of the most abundant tRFs have been estimated to be 100–100,000 molecules per sperm [15], raising the question of the extent to which sperm RNAs contribute to early embryonic gene expression and offspring phenotypes. Second, in the microinjection studies investigating the role of sperm sRNAs in paternal inheritance, non-physiological levels of sRNAs were injected into embryos. Although genetic manipulation of sRNA pathway components demonstrated a role of sperm sRNAs in regulating embryonic development [21], the functional consequences of the delivery of physiological levels (amount of RNA in a single sperm) of environment-responsive sperm sRNAs in early embryos remain to be determined. The microinjection studies do, however, provide evidence that changing the levels of RNA or stability of RNAs has profound effects on embryonic gene expression and offspring health. Third, the sRNA payload of sperm that fertilize the oocyte has not been examined. The current understanding of sperm sRNA payload comes from mature epididymal sperm or ejaculated human sperm; however, sperm continue to mature and undergo changes (e.g., capacitation, acrosome reaction) as they travel through the female reproductive tract. It is unclear how acrosome reaction, wherein sperm shed their cytoplasm and plasma membrane influences sperm sRNA levels. Nonetheless, at least a subset of sperm sRNAs are enriched in sperm nucleus [17,60] and can be delivered to the oocyte at fertilization. Finally, as discussed above, without a mechanism to amplify sperm-delivered sRNAs during embryonic development, sperm sRNAs will be diluted with every cell division. Therefore, a major gap in our understanding of the mechanism of sRNA-mediated intergenerational inheritance is how sperm sRNAs modulate persistent changes in gene expression to give rise to a phenotype in adult offspring.

To understand the mechanism of sRNA-mediated inheritance of paternal environmental effects, three major questions need to be addressed: (1) how do environmental conditions, such as a low protein diet or an early life trauma, affect sRNA levels in sperm, (2) to what extent do sperm sRNAs contribute to the early embryonic RNA repertoire and what roles do they play in early embryonic development, and (3) how do sRNA-regulated changes in preimplantation embryos lead to altered phenotypes in adults? Moreover, a deeper understanding of the underlying mechanism of intergenerational inheritance is required to develop hypotheses and design appropriate experiments to study the mechanism of transgenerational inheritance.

## Figures and Tables

**Table 1:** Overview of the different sRNA-sequencing methods.

Sequencing Methods	Pretreatment of RNA	Sequencing of RNA with 5′OH	Sequencing of RNA with 2′–3′ Cyclic Phosphate/3′ Phosphate	RT through Modified RNA Nucleotides	Small RNA Classes Preferentially Sequenced
OTTR-seq	−/+ PNK	Yes	Yes	Yes	tRFs, rRNA fragments, miRNAs, and other non-modified small RNA classes
DM-tRNA	AlkB (wild type and D135S mutant)	No	No	Yes	tRFs, miRNAs, and other non-modified small RNA classes
ARM-seq	AlkB	No	No	No	tRFs, miRNAs, and other non-modified small RNA classes
PANDORA-seq	T4 PNK, AlkB	Yes	Yes	No	tRFs, rRNA fragments, miRNAs, and other non-modified small RNA classes
Ligation-dependent	No	No	No	No	miRNAs, and other non-modified small RNA classes

## Data Availability

Not applicable.
